# Estimating the false-negative test probability of SARS-CoV-2 by RT-PCR

**DOI:** 10.2807/1560-7917.ES.2020.25.50.2000568

**Published:** 2020-12-17

**Authors:** Paul S Wikramaratna, Robert S Paton, Mahan Ghafari, José Lourenço

**Affiliations:** 1 These authors contributed equally to this article and share first authorship; 2 Independent Researcher, London, United Kingdom (DPhil (Zoology) Oxon); 3 Department of Zoology, University of Oxford, Oxford, United Kingdom

**Keywords:** RT-PCR, COVID-19, SARS-CoV-2, testing, accuracy

## Abstract

**Background:**

Reverse-transcription PCR (RT-PCR) assays are used to test for infection with the SARS-CoV-2 virus. RT-PCR tests are highly specific and the probability of false positives is low, but false negatives are possible depending on swab type and time since symptom onset.

**Aim:**

To determine how the probability of obtaining a false-negative test in infected patients is affected by time since symptom onset and swab type.

**Methods:**

We used generalised additive mixed models to analyse publicly available data from patients who received multiple RT-PCR tests and were identified as SARS-CoV-2 positive at least once.

**Results:**

The probability of a positive test decreased with time since symptom onset, with oropharyngeal (OP) samples less likely to yield a positive result than nasopharyngeal (NP) samples. The probability of incorrectly identifying an uninfected individual due to a false-negative test was considerably reduced if negative tests were repeated 24 hours later. For a small false-positive test probability (<0.5%), the true number of infected individuals was larger than the number of positive tests. For a higher false-positive test probability, the true number of infected individuals was smaller than the number of positive tests.

**Conclusion:**

NP samples are more sensitive than OP samples. The later an infected individual is tested after symptom onset, the less likely they are to test positive. This has implications for identifying infected patients, contact tracing and discharging convalescing patients who are potentially still infectious.

## Introduction

Currently, most SARS-CoV-2 infected individuals are identified by successful amplification of the virus from nasopharyngeal or oropharyngeal swabs using a reverse-transcription PCR (RT-PCR) assay. These tests are highly specific but there are many reasons why sensitivity is imperfect [1]. Indeed, multiple studies have observed negative RT-PCR results on at least one occasion for SARS-CoV-2 infected individuals [1-8]. Such false-negative results have implications for correct diagnosis [9] and subsequent community transmission [10], and for control initiatives, not only during emergence but also in any subsequent waves of transmission. Successful attempts to test and trace resurgent outbreaks require a better understanding of how test results can confirm or rule out whether an individual is infected with the virus, but also need to be taken in context with other available information (such as the timing and type of test performed).

A series of previous studies have described cohorts of tested individuals. Ai and colleagues [2] retrospectively considered 1,014 infected patients, of whom 413 (41%) tested negative by RT-PCR at initial presentation. Xie et al. [1] similarly considered 167 infected patients of whom, five (3%) tested negative by RT-PCR at initial presentation. Fang et al. [3] found that RT-PCR was only able to identify 36/51 (71%) of SARS-CoV-2 infected patients when using swabs taken 0–6 days after symptom onset. Liu et al. [8] and Zhao et al. [7] demonstrated that the proportion of positive tests among infected patients reduced with each week after symptom onset, and Luo et al. [11] reported that the initial sensitivity of oropharyngeal swabs in secondary contacts was 71%. Meanwhile, in a study of 213 patients, Yang et al. [4] found lower positive test rates from oropharyngeal swabs (24%) compared with nasopharyngeal swabs (57%). Although these particular studies relate to longitudinal testing of infected patients, the data are not disaggregated per patient. Some authors have, however, presented sequential test data from individual patients [5,6,12].

We aim to estimate the likelihood of correctly identifying the SARS-CoV-2 infection status of an individual by RT-PCR tests using sequential test data from individual cases. These data are used to characterise how the probability of a false-negative test result depends on the number of days between symptom onset and the test date, and how this is affected by the type of swab used (oropharyngeal or nasopharyngeal). We also estimate the number of false negatives in different cohorts of tested individuals under the assumption that they are only tested once, and assess the effect of test specificity on these results.

## Methods

### Data sources

Data were obtained from tables and figures in previously published studies and preprints that were available as of 15 June 2020. Our research was conducted at the beginning of the coronavirus pandemic when only a small number of peer-reviewed publications and datasets were available in PubMed. We therefore broadened our sources to include the medRXiv pre-print server using the search terms ‘RT-PCR’ and ‘COVID-19’ on 13 April 2020 (yielding 57 pre-prints) and on 15 June 2020 (yielding 201 pre-prints)*.* To qualify for inclusion, studies had to report extractable results for longitudinal RT-PCR tests from individual patients who tested positive for SARS-CoV-2 at least once with a well-defined date of symptom onset. The timing of swab collection had to be stated as the number of days after the patient became symptomatic. We identified seven studies that met these criteria and reported nasopharyngeal swab results [5,12-17] with two also reporting results for oropharyngeal swabs in the same patients [5,12]. Several studies with longitudinal patient data, including [6,18,19], only vaguely described the sampling location (e.g. upper respiratory tract) or did not make clear which results belonged to which patient. Other types of sample - such as sputum and saliva - were not considered as these collection methods are different to swabs. The seven studies provided data from 787 tests on 95 patients using nasopharyngeal or oropharyngeal swabs. Summary information on the included studies is presented in the Table.

**Table:** Summary of the data sources used in this study

Publication	Country where testing was performed	Target gene for RT-PCR test	Swab type(s) and (number)	Infection definition and case description	Number of cases included (reported in the study)
Danis et al. [13]	France	Not specified	Nasopharyngeal (38)	All patients were isolated in hospital and were RT-PCR confirmed. Five of the six presented symptoms. One patient was only confirmed using endotracheal aspirate samples and was excluded from the analysis.	4 (6)
Kujawski et al. [12]	United States	3 targets on N	Nasopharyngeal (86) and oropharyngeal (90)	Seven of 12 patients were hospitalised with five remaining at home. All were RT-PCR confirmed. One patient was excluded due to a poorly defined symptom start date.	11 (12)
Lescure et al. [17]	France	RdRp-IP1 and RdRp	Nasopharyngeal (42)	All five patients were admitted to hospital and were RT-PCR confirmed.	5 (5)
Seah et al. [14]	Singapore	Not specified	Nasopharyngeal (132)	All 17 patients were recruited while in hospital and were RT-PCR confirmed.	17 (17)
Wyllie et al. [15]	United States	Multiple targets on N (CDC assay)	Nasopharyngeal (53)	All patients were RT-PCR hospital patients with symptoms. Of the 44 patients, 22 had longitudinal NP testing time series.	22 (44)
Young et al. [16]	Singapore	N, S and ORFab1	Nasopharyngeal (230)	All patients were admitted to hospital and RT-PCR confirmed.	18 (18)
Zou et al. [5]	China	N and ORF1b	Nasopharyngeal (61)^a^ and oropharyngeal (55)	All cases were RT-PCR confirmed. One patient was asymptomatic.	17 (18)
**Total **					**95 (120)**

CDC: Centers for Disease Control and Prevention; NP: nasopharyngeal; OP: oropharyngeal.

^a^ This study used a combination of nasopharyngeal and mid-turbinate swabs.

### Data analysis

#### Estimating reverse transcription-PCR assay sensitivity

Data were analysed using binomially distributed (logit-link) generalised additive mixed models (GAMM) with the package ‘mgcv’ [20] version 1.8-31 in the statistical software R version 3.6.3 (R foundation, Vienna, Austria) [21]. The effect of the number of days since symptom onset was modelled as a continuous smooth function (cubic regression splines), while swab type and data source were included as two-level categorical variables. Random effects were included in the form of patient-specific smooth functions, modelling between-patient differences on the probability of returning a positive test with time. All the models we examined included this random effect as patient samples were pseudo-replicated by design. Models were compared in a stepwise down procedure from the most complex structure using Akaike Information Criterion (AIC). The difference in AIC values (ΔAIC) were calculated with respect to the lowest AIC value. Detailed methodology is available in Supplementary File 1. These results are reported together with 95% confidence intervals.

#### Estimating the number of false negatives in a cohort of tested individuals

Results from Bi et al. [22] suggest that the probability of an infected individual having a positive RT-PCR test for SARS-CoV-2 after a given number of days since symptom onset follows a gamma distribution with shape 2.12 and rate 0.39 (see both Figure 2 and Table S2 in Bi et al. [22]). We used these data with our results on RT-PCR sensitivity and applied Bayes’ Theorem to recover the distribution of the time from symptom onset to RT-PCR test (see Supplementary File 1).

We then integrated this distribution over time from symptom onset to time of test in order to calculate the population average false negative test probability (i.e. taking account of the fact that the false negative test probability depends on time from symptom onset). We used this estimate alongside a realistic range of false positive test probabilities to illustrate by how much the true number of infections in a cohort of tested individuals can differ from the number of positive tests (see Supplementary File 1 for more details), based on data from the United Kingdom (UK) and South Korea as of 20 March 2020 [23] (UK: 5.1% positive (3,277/64,621); South Korea: 2.7% (8,652/316,664).

#### Reproducibility

To aid reproducibility, we have provided the main results, R scripts and data used as Supplementary Files 2, 3 and 4, respectively.

#### Ethical statement

No ethical approval was required for this meta-analysis of publicly available data.

## Results

Our most complex model included two fixed effects (a smooth effect of day and a different intercept for each swab type) as well as a random intercept for each study and each patient (model AIC = 805.79). Removing the random intercept for study was supported (AIC = 805.31, ΔAIC = -0.47), suggesting that the baseline probability of detection was consistent across different cohorts, test targets and sample processing procedures. Excluding swab type was not supported (AIC = 813.15, ΔAIC = 7.83), nor was excluding the effect of days since symptom onset (AIC = 974.30, ΔAIC = 168.99). The final model structure with the highest support contained the fixed effect of swab type, the effect of time since symptom onset and a random intercept for each patient. The full model output is given in Supplementary File 1.

**Figure 1:**
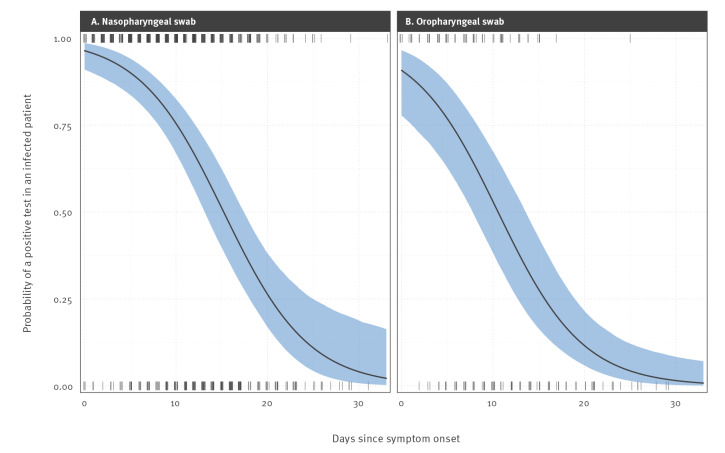
Impact of time post symptom onset on positive RT-PCR test result probabilities for SARS-CoV-2 infected individuals^a^ using (A) nasopharyngeal swabs and (B) oropharyngeal swabs, 2020 (n = 95)

GAMM: generalised additive mixed models; SARS-CoV-2: severe acute respiratory syndrome coronavirus 2.

^a^ Data were obtained from published studies on patients from China, United States, France and Singapore. Tick marks denote positive (top) and negative (bottom) tests (jittered on the x-axis for visual purposes). The black line shows the binomially distributed GAMM model fit, and the blue shading indicates 95% confidence intervals on the fixed effects. Visualising the impact of time to test on false-negative test probabilities.

Oropharyngeal swabs taken immediately upon symptom onset were predicted to be 5.64% less likely to yield a positive result than a nasopharyngeal swab (logit-scale effect size −1.00 (95% CI: −1.54 to 0.46)). The probability of a positive test decreased with number of days past symptom onset. For a nasopharyngeal swab, the percentage chance of a positive test declined from 96.40% (95% CI: 90.98 to 98.6) on day of symptom onset to 75.47% (95% CI: 66.88 to 82.51) on day 10 since symptom onset (SSO), and only a 3.30% (95% CI: 0.53 to 17.90) chance of a positive result on day 31 SSO. For an oropharyngeal swab, the probabilities were 90.76% (95% CI: 77.84 to 96.52), 53.00% (95% CI: 38.27 to 67.46) and 1.23% (95% CI: 0.18 to 7.86) for day of symptom onset and days 10 and 31 SSO, respectively. The model fit is shown in Figure 1 and the underlying quantitative results (data) are available in Supplementary File 2.

As shown above, the probability of a false-negative test result depends on the number of days since symptom onset. This means that simple reports of positive and negative test counts among individuals who are only tested once will underestimate the true number of positive tests in a cohort. We can illustrate the potential impact this has on average false-negative test probabilities by assuming that time from symptom onset to testing follows a gamma distribution.

**Figure 2:**
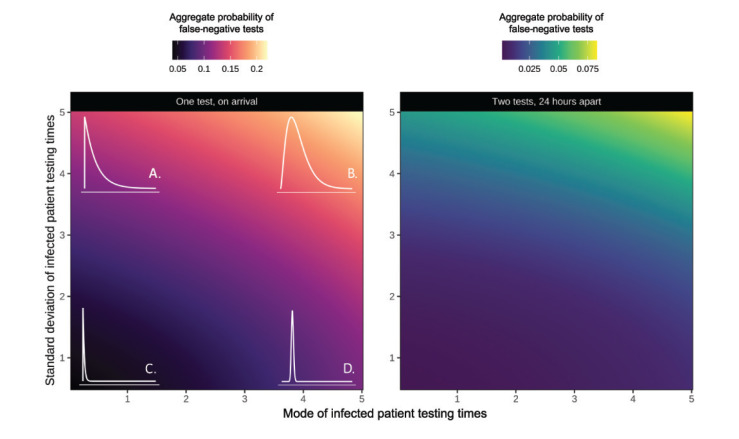
Aggregate probability of false-negative tests for gamma-distributed cohorts of individuals tested for SARS-CoV-2 infection^a^ including a scenario where infected individuals are tested (A) mostly early but with a long-tail of individuals taking a long time to be tested, (B) mainly later, with a similarly long tail, (C) consistently early, (D) consistently tested later, 2020

Figure 2 explores how varying the shape and rate of this gamma distribution affects the average false-negative test probability in a cohort, and highlights that in scenarios where infected individuals are typically tested late, false-negative test probability can be four times larger than when patients are typically tested early. We also show that the probability of incorrectly identifying an individual as uninfected due to a false-negative test considerably reduces if all negative tests are repeated 24 hours later. We note that the realised error rate (the actual proportion of false-negative tests) will be proportional to the underlying prevalence of infection, and that the probability of a false-negative will only equal the proportion of negative tests (as there will be no true negatives from uninfected individuals) if everyone in the cohort is infected.

SARS-CoV-2: severe acute respiratory syndrome coronavirus 2.

^a^ Data were obtained from published studies on patients from China, United States, France and Singapore.

Each point on the surface is a result for a unique parameterisation of the distribution. The x-axis shows the mode of the testing time gamma distribution and the y-axis the standard deviation. The left panel shows the error rate for one test, while the right panel shows the error rate for two tests taken 24 hours apart. Each colour denotes a different aggregate probability of obtaining a false-negative test, with the lighter colours indicating a higher probability. Illustrative distributions are drawn in the corresponding corners of the first panel (these are the same in the second panel), with subplots A-D showing illustrative examples of four extremes.

### Estimating the number of false negatives in a cohort of tested individuals

**Figure 3:**
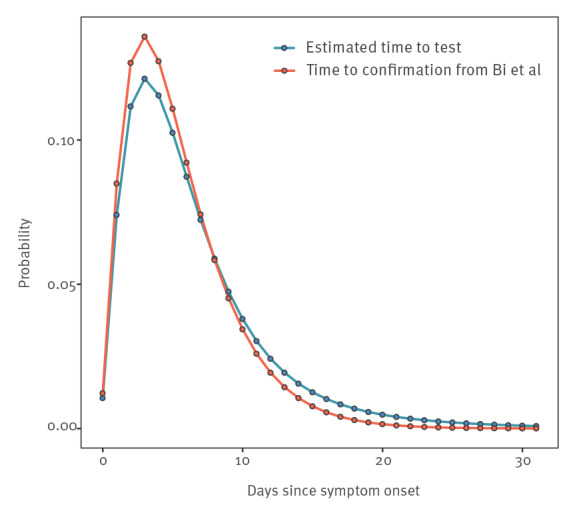
Comparison of the discretised distributions of estimated time from symptom onset to confirmation among SARS-CoV-2 symptomatic individuals from the data in Bi et al. (2020) [22], China (n = 391), and these data combined with the false negative test probability results from the present study^a^, 2020 (n = 95)

We estimated the distribution of time from symptom onset to taking a test by coupling an estimate of the time from symptom onset to positive test result [22] with our results on false negative test probabilities. Figure 3 shows the result, which has a heavier tail than the original distribution from Bi et al. [22] because the false-negative test probability increases with time. We used this result to calculate the average false negative test probability for a population whose time from symptom onset to test is as shown in Figure 3 and now demonstrate how this result (a false-negative test probability of 16.7%), together with assumptions about the false-positive test probability, may affect testing outcomes in practice.

SARS-CoV-2: severe acute respiratory syndrome coronavirus 2.

^a^ Data were obtained from published studies on patients from China, United States, France and Singapore.

The data from Bi et al. [22] (red line) and the distribution of time from symptom onset to test that we estimated, combined with our estimate of the false-negative test probability from nasopharyngeal swabs (blue line), assuming false-positive tests are impossible (see Supplementary File 1).

**Figure 4:**
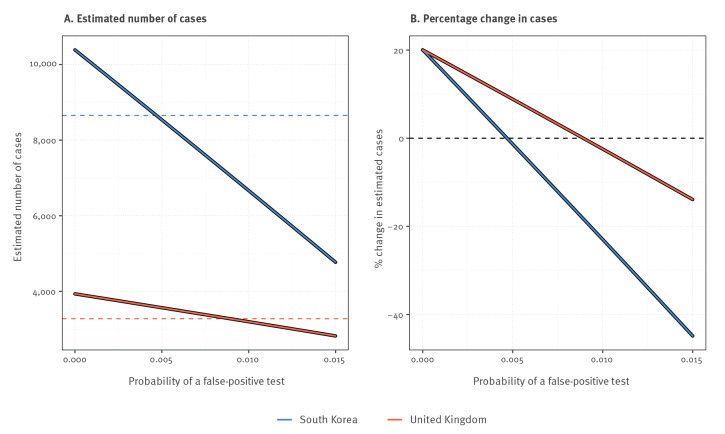
How the probability of obtaining a false-positive RT-PCR test result for SARS-CoV-2 infections changes (A) the estimated number of cases and (B) the estimated percentage change in number of cases for South Korea (n = 316,644) and United Kingdom (n = 64,621), 20 March 2020

Figure 4 shows that when the probability of a false-positive test is small, the estimated number of infections among those tested is increased by around 30%. This estimate decreases linearly as false-positive test probability increases. Thus, for a critical (yet small) false-positive test probability value, there will be more false positives than false negatives. Moreover, the false-positive test probability has a bigger impact when the percentage of originally positive tests is smaller (this follows directly from the underlying derivations (Supplementary File 1)). Finally, if the false-positive test probability is between 0.5% - 1%, the true prevalence among those tested can be lower than suggested by the naive count of positive tests.

SARS-CoV-2: severe acute respiratory syndrome coronavirus 2.

Dashed line corresponds to number of positive tests conducted as of 20 March 2020.

It is important to stress that this exercise is illustrative rather than assertive and relies on broad assumptions (e.g. all individuals are only tested once, that the distribution of time from symptom onset to test is as we have estimated and that all those tested are symptomatic), any or all of which are likely to be violated in these datasets. In other words, this exercise merely highlights the potential impact of accounting for the false-negative and false-positive test probabilities.

Nevertheless, Figure 4 illustrates three important things: (i) that for a zero or very small false-positive test probability, the true number of infected individuals among those tested will be substantially larger than the number of positive tests; (ii) that increasing false-positive test probabilities decrease these estimates until they eventually become negative (even for quite small values of the false-positive test probability); and (iii) that such decreases are more severe in situations where the apparent prevalence among those tested is lower.

## Discussion

Testing from a single nasopharyngeal or oropharyngeal swab by RT-PCR is not guaranteed to yield a (true) positive result for SARS-CoV-2 infection, and the probability of obtaining a true positive result decreases with time from symptom onset. In other words, the longer the time from symptom onset to a suspected case being tested, the higher the likelihood of a false-negative result. Repeat testing of suspected but RT-PCR negative infections may not always be feasible, for example when testing capacity is limited, but our results suggest that repeat testing drastically decreases the chances of failing to identify infected individuals.

Meanwhile, early estimates for case and infection fatality rates of SARS-CoV-2 relied on perfect test sensitivity among international travellers [24,25], risking an upward bias by not accounting for the possibility of false-negative tests.

Contrastingly, we showed how even small false-positive test probabilities can have an opposite effect on any assessment of the true number of infections in a tested cohort and bias case and infection fatality risk estimates in the opposite direction. Better understanding of the false-positive test probability, and accounting for precisely when and how individuals have been tested would therefore improve the quality of any estimates that rely on the number of positive tests in a cohort of tested individuals.

Our results based on available data from early in the pandemic, have implications for SARS-CoV-2 testing strategies put in place at the time. RT-PCR testing regimes varied considerably between countries, determined by policy decisions, testing capacity and perceived incidence. Some countries have opted (or, rather, were able) to test large portions of the population, including those who are asymptomatic or self-isolating with mild symptoms. In countries such as South Korea, where testing has been thorough [26,27], the distribution of test timing is crucial. If many of those tested were infected days or weeks prior to testing but only had mild or asymptomatic infections (and therefore did not present for treatment), they would be more likely to return a false-negative result. While RT-PCR testing of key workers is of great importance (particularly those working with vulnerable groups), our results suggest that there may be little benefit to testing indiscriminately; in fact, conducting a single test on someone who had symptoms 10 days ago will have a nearly 25% chance of being a false-negative (using a nasopharyngeal swab, or 47% for an oropharyngeal swab). As a means of determining population level exposure to SARS-CoV-2, serological tests of known specificity and sensitivity are far more likely to provide an accurate profile. If specificity and sensitivity are known, then recovery of the likely level of population exposure is possible, even if it is not possible to determine which individuals have been exposed. This, however, would assume that all those infected would go on to develop and maintain detectable serological responses, which may not always be the case in mild infections, those asymptomatic, or across all age groups [28-33].

In almost all countries, tests will be conducted on patients presenting with symptoms at a hospital in order to streamline treatment and prevent further infection. We do not suggest that the problem of false negatives is under appreciated by medical professionals; it is presently recognised in guidelines from both the World Health Organization (WHO) [34] and the European Centre for Disease Prevention and Control (ECDC) [35] that a single negative test is insufficient to rule out infection, with discharge criteria stating that a patient should only be released if two repeat tests return negative results. Early in the outbreak, clinicians used a chest computed tomography scan to look for evidence of SARS-CoV-2 in symptomatic patients who returned a negative result, minimising the risk of false negatives [2,36].

We also note that RT-PCR tests will return positive results even if the virus is inert – only by culturing the sample is it possible to verify that a patient is actually infectious. To date, the available information suggests that it is possible for individuals with mild disease to shed for at least 18 days [37], and those with severe disease for at least 20 [18]. However, evidence is mounting that successful culture of the virus is associated with high viral loads and perhaps not yet having a detectable serological response [6,18,38], which suggests that quantitative estimates of (still detectable) viral load and/or seroconversion could be used as tools to safely discharge infected individuals from quarantine or hospital [18]. In light of this evidence, our study cannot readily assist with such decisions, not least because the higher false-negative test probabilities we report with time SSO are themselves almost certainly related to lower viral loads over time. Thus, further research involving quantitative estimates of how viral load changes with time since symptom onset would be useful. Repeat measurements and calculation of viral load in infected individuals allow a more precise interpretation of the RT-PCR results, specifically whether a negative or high cycle threshold (CT) result is consistent with the previous trend in that particular patient or is anomalous, and so determine how likely the individual is to still be infectious. Based on limited data available early in the pandemic, our results nonetheless suggest that multiple negative tests would be consistent with the viral load having reduced to a sub-infectious level, but a single negative test could represent only a spurious test result.

Major implications of our results relate directly to contact tracing of infected individuals. Since it is possible for infected individuals to test negative for infection soon after the development of symptoms, it is theoretically incorrect to assume that a single negative test in a suspected symptomatic index case rules out infection in their close contacts who may be better advised, in a best-case scenario, to self-isolate regardless. It may therefore be prudent to assume that contacts of suspected infected individuals may be infected themselves and isolate, regularly testing them until either there is firm evidence that the putative index case was not actually infected (or at least infectious) at the time of the contact, or assess if the incubation period for the contact has passed without evidence that the contacts have themselves been infected.

### Limitations

First, more data exist than we have been able to analyse. Many of the studies cited here ([1-4,6-11,18,19,39]) have longitudinal data from more patients but were not publicly available at the time of writing, are not disaggregated by swab type, or do not have clear data on symptom onset. Inclusion of these data would provide superior estimates, in particular if they are disaggregated into tests from different samples via different routes for the same patient. Moreover, explicit reporting of test dates in all patients (and not just those who test positive) would be especially useful to any subsequent similar analyses for SARS-CoV-2 or other emerging viruses. We thus advocate for such data to be made available more readily in publications and preprints. We also note that the data used here comes from a mix of already published papers, and not yet peer-reviewed preprints.

Second, we attempted to account for possible differences among laboratories performing RT-PCR tests, and although we did not find any evidence in favour of this being relevant, there is not enough evidence to rule it out. There may be variations in terms of the gene targeted or method of RT-PCR performed, which we have not been able to consider due to lack of available data (e.g. some target / assay combinations may be more sensitive than others [40,41]). We have also assumed that all patients have been correctly identified as infected in each study, and that test specificity is perfect. Test specificity is likely to be extremely high, but not 100% and may similarly vary with assay type [42].

Third, we have attempted to account for possible differences in patient sensitivity to the tests. In reality, one might expect this to be related to either the underlying severity of the infection (perhaps a higher chance of detection when the disease is more severe) or viral load (higher chance of detection with a higher viral load), neither of which we have been able to assess with the currently available data. Furthermore, the data we used are from symptomatic patients, most of whom required hospital treatment, and it is possible that the RT-PCR test is less sensitive in asymptomatic individuals (not least because there is no onset of symptoms and it is therefore unclear from which baseline test sensitivity should be measured), or those with mild symptoms. A recent Italian study offered evidence that, among those testing positive, viral loads were equivalent in symptomatic and asymptomatic individuals [43]. This does not show, however, that viral loads are the same in both groups, but that they are equivalent conditional on a positive test, which is what we might expect if the probability of a positive test is indeed linked to viral load. If this is true, then it could be that many asymptomatically infected individuals are asymptomatic because their immune responses keep viral replication in check early on and so viral loads sufficient to result in a positive test may not be achieved. However, typically low viral loads in asymptomatic individuals are hard to reconcile with their apparent transmission potential [44]. Better understanding of the sensitivity of the test in asymptomatic individuals is of paramount importance, but this was impossible to assess with the currently available data.

Fourth, we did not have access to testing data from the pre-symptomatic period of infection. The high detection probability at symptom onset suggests that viral load could be high during the incubation period, particularly just before symptom onset. We would also expect that the chances of detecting infection on the day of exposure is next to zero, but the detection probability between exposure and before symptom onset will likely be contingent on a number of factors including the initial infectious dose of SARS-CoV-2 and the length of the incubation period. More data are urgently needed to provide information on viral load development between infection and symptom onset, particularly given the estimated transmission potential in this period [39,45].

Finally, when estimating the true number of positives in a cohort of tested individuals we have to assume that the distribution of the time to test is the same as we inferred from our results in the present study, and the distribution of time to confirmation in Guangdong [22]. Even if this distribution is broadly representative from country to country, it may not be consistent over time. For example, as testing capacity reaches its limits, time to test may increase and so too the probability of a consequent false-negative (or vice versa as testing capacity is enhanced). These particular results should therefore be taken as indicative rather than authoritative. Furthermore, these results only relate to the cohort of tested individuals rather than the population at large; they say nothing about the prevalence of the virus among those not tested. That said, individual hospitals, testing centres or studies will know the timings of their tests and can use such information in conjunction with the quantitative findings that we make available in the Supplementary files to assess how likely any one test is to represent a false-negative.

### Conclusion

This work has advanced our understanding of the considerable effect that false negative RT-PCR tests can have on the identification of SARS-CoV-2 infected individuals. We have demonstrated the sensitivity of population prevalence estimates to erroneous test results, and caution that single negative tests should not be overinterpreted. This is particularly pertinent when tests are used to determine whether healthcare staff and carers are safe to work with those most vulnerable to disease, or whether to isolate contacts of suspected infections. As nations began lifting strict social distancing measures and returning to some semblance of normality after the first epidemic wave, being able to dependably contact trace and test new infections became critical to prevent resurgence. This work, performed in June 2020 at a time where most nations were experiencing their first COVID-19 epidemic wave, suggests caution should be used when interpreting a single, SARS-CoV-2 RT-PCR test result.

In conclusion, we demonstrate how the sensitivity of the RT-PCR assay for detecting SARS-CoV-2 infection depends on the time from symptom onset in symptomatic individuals, and show how nasopharyngeal swabs appear more sensitive than oropharyngeal swabs. In the absence of other testing procedures, this dependence on time since onset has implications for clinical decisions about treatment, and control / contact tracing decisions about who needs to be quarantined or can be released safely into the community. We also illustrate how, assuming that the false-positive test probability is negligible, the positive test count underestimates the number of infected individuals count in a cohort of tested individuals, which in turn has implications for estimates of case and infection fatality rates in the wider population.

## Supplementary Data

143000 Supplementary Material 1

## Supplementary Data

2000 Supplementary Material 2

## Supplementary Data

15000 Supplementary Material 3

## Supplementary Data

5000 Supplementary Material 4
