# Supplementary material for: Estimating the false-negative test probability of SARS-CoV-2 by RT-PCR
Source: Euro Surveill. 2020 Dec 17;25(50):2000568. doi: 10.2807/1560-7917.ES.2020.25.50.2000568 (PMC7812420; doi:10.2807/1560-7917.ES.2020.25.50.2000568)
Supplement: Supplementary Material 2 [file 20-00568_WIKRAMARATNA_SuppFile2_results.zip › Disclaimer.docx]

This supplementary material is hosted by *Eurosurveillance*as supporting information alongside the article 'Estimating the false-negative test probability of SARS-CoV-2 by RT-PCR', on behalf of the authors, who remain responsible for the accuracy and appropriateness of the content. The same standards for ethics, copyright, attributions and permissions as for the article apply. Supplements are not edited by *Eurosurveillance* and the journal is not responsible for the maintenance of any links or email addresses provided therein.
